# The role of MorI/MorR quorum sensing in *Methylobacterium oryzae* CBMB20: modulating bacterial functions for enhanced adaptability

**DOI:** 10.1128/spectrum.02117-25

**Published:** 2025-09-12

**Authors:** Qiying Deng, Yue Zheng, Huagui Gao, Haofang Wu, Enyu Shi, Mengmeng Cheng, Huishan Wang, Lisheng Liao

**Affiliations:** 1 Guangdong Province Key Laboratory of Microbial Signals and Disease Control, Integrative Microbiology Research Center, South China Agricultural University 12526, Guangzhou, China; 2 CAS Key Laboratory of Urban Pollutant Conversion, Institute of Urban Environment, Chinese Academy of Sciences 85406 , Xiamen, China; 3 State Key Laboratory of Marine Environmental Science and College of the Environment and Ecology, Xiamen University 12466, Xiamen, China; 4 College of Agriculture and Biology, Zhongkai University of Agriculture and Engineering, Guangzhou, China; Emory University School of Medicine, Atlanta, Georgia, USA

**Keywords:** *Methylobacterium oryzae*, quorum sensing, AHLs, plant growth promotion, bioinoculant, transcriptome analysis

## Abstract

The quorum-sensing (QS) system in *Methylobacterium oryzae* CBMB20, an endophytic bacterium associated with rice (*Oryza sativa* L.), plays a critical role in regulating bacterial behaviors essential for plant growth promotion and adaptation. This study aimed to elucidate the functional role of the MorI/MorR QS system in *M. oryzae* CBMB20 and its potential application as a bioinoculant. We identified and characterized two QS signals, 3-OH-C12-HSL and 3-oxo-C12-HSL, synthesized by the MorI enzyme. The MorR receptor was found to preferentially respond to 3-OH-C12-HSL, indicating a high degree of specificity in QS signaling. The deletion mutants of *morI* and *morR* exhibited significant changes in exopolysaccharides (EPS), motility, and methanol utilization, suggesting that the MorI/MorR system is crucial for bacterial survival and adaptation. Transcriptome analysis revealed that MorR acts as a repressor, controlling the expression of numerous genes, many of which are upregulated upon its deletion. Our findings highlight the multifaceted role of the MorI/MorR QS system in *M. oryzae* CBMB20, influencing key biological functions such as EPS production, motility, and methanol utilization. The modulation of these traits through QS could enhance the bacterium’s effectiveness as a bioinoculant for promoting plant growth. This study contributes to the understanding of how QS systems can be harnessed to improve the efficacy of plant growth-promoting bacteria in agricultural settings, offering insights into the potential for genetic manipulation to optimize bioinoculant performance.

**IMPORTANCE:**

This study provides critical insights into microbial communication by functionally characterizing the MorI/MorR quorum-sensing (QS) system in *Methylobacterium oryzae* CBMB20, a plant-beneficial methylotroph. We identify 3-OH-C12-AHL as a key long-chain signal governing exopolysaccharides biosynthesis, swimming motility, and methanol metabolism traits pivotal for host colonization. These findings not only elucidate novel regulatory mechanisms in plant-associated bacteria but also pave the way for engineering QS-driven strategies, such as synthetic consortia or targeted microbiome interventions, to enhance sustainable agricultural practices.

## INTRODUCTION

Quorum sensing (QS) is a unique mechanism for microbial cell-to-cell communication that can regulate a range of community behaviors (1, 2). LuxR-LuxI-type QS circuits, common to many species of *Proteobacteria* (3), involve LuxI-type synthases that synthesize acyl-homoserine lactones (AHLs). These AHLs are recognized by complementary LuxR-type receptors (1–4), which then interact with specific DNA sequences to modulate the transcription of target genes (1–4). AHL QS serves as a pivotal regulatory system that facilitates coordinated behavior among bacteria (5). AHL-mediated QS is a critical regulatory system that facilitates synchronized behaviors among bacteria, and in mutualistic contexts, it allows for adaptive responses to the host’s environment, bolstering colonization, persistence, and beneficial effects within the host (6).

*Methylobacterium* is generally known by the term pink pigmented facultative methylotrophic, able to use one-carbon compounds, such as methanol excreted by plants (7). The *Methylobacterium* genus, also known for its mutualistic relationships, constitutes a substantial component of plant microbiomes and is categorized as plant growth-promoting bacteria (PGPB) (7–10). In the mutualistic relationship between *Methylobacterium* and plants, *Methylobacterium* enhances plant growth rates by producing cytokinins, while simultaneously utilizing the methanol released by the plants as a nutritional source (10–12).

Studies have shown that AHL QS in *Methylobacterium* species regulates key group behaviors essential for survival and environmental interaction (13–17). For instance, *Methylobacterium extorquens* AM1 employs two LuxI homologs to produce AHLs of varying chain lengths under different growth conditions (13). Additionally, a truncated LuxI homolog named *tslI* controls the synthesis of AHLs and positively regulates the production of exopolysaccharides (EPS), potentially contributing to biofilm formation (14). *Methylobacterium mesophilicum* SR 1.6/6 connects AHL presence to the regulation of genes pivotal for methanol transformation, a critical aspect of methylotrophic metabolism (15). *Methylobacter tundripaludum* 21/22 uses QS to regulate the production of the metabolite tundrenone (17.18). Recently, a highly homologous QS system in *Methylobacterium fujisawaense* DSM5686 was found, in which the AHL signal 3R-OH-5Z-C12:1-HSL activates a LuxR-family regulator, inhibiting swarming motility (18). QS in *Methylobacterium* species is a multifaceted communication system that plays a significant role in regulating behaviors that are important for bacterial adaptation and interaction with their surroundings.

*Methylobacterium oryzae* CBMB20, an endophytic bacterium living in symbiosis with rice (*Oryza sativa* L.), influences the plant’s photosynthetic traits, volatile organic compound emissions, and ethylene metabolism (19). This bacterium contains a set of *luxI*/*luxR* homologs, designated as *morI* and *morIR* (JGI/IMG Locus Tag Ga0069285_115886 and Ga0069285_115887). The study described here performed a functional analysis of the MorI/MorR QS system in strain CBMB20. Data in this study suggested that MorR might function as a repressor of QS in the control of motility, biofilm formation, and oxidative stress response. These findings suggest that AHL QS in *M. oryzae* CBMB20 could be utilized in plant growth promotion and as a bioinoculant for the phyllosphere.

## RESULTS

### Purification and structural determination of AHL produced by the *M. oryzae* CBMB20

In pursuit of purifying and identifying the signals produced by MorI, we engineered a MorI signal-specific reporter. Sequence alignment revealed a conserved LuxR binding motif within the MorI promoter (Fig. S1). Given that LuxR-LuxI-like AHL regulatory circuits often autoregulate AHL synthesis, we constructed a MorR-PmorI-mCherry reporter plasmid (pDQY1) as previously described (20). This involved fusing the *morI* promoter to the *mCherry* gene, along with the MorR coding sequence, into the expression vector pBBR1-MCS5 (Fig. S2A). The construct was then introduced into the non-QS strain *Pseudomonas putida* F1, yielding the reporter strain PDQY1. The design was based on the premise that the AHL synthase gene morI is regulated by quorum sensing, with signal generation triggered by the MorR receptor, which is a signal-bound LuxR-type regulator. As a control, we constructed pDQY2 (Fig. S2B), which contained the *morI* promoter-controlled *mCherry* but lacked *morR*. Bioassay results confirmed the reporter strain PDQY1 had robust fluorescence in response to *M. oryzae* CBMB20 culture extracts. Expression of *mCherry* was not influenced in *P. putida* PDQY2, validating its utility for CBMB20 signal detection (Fig. S2C).

**Fig 1:**
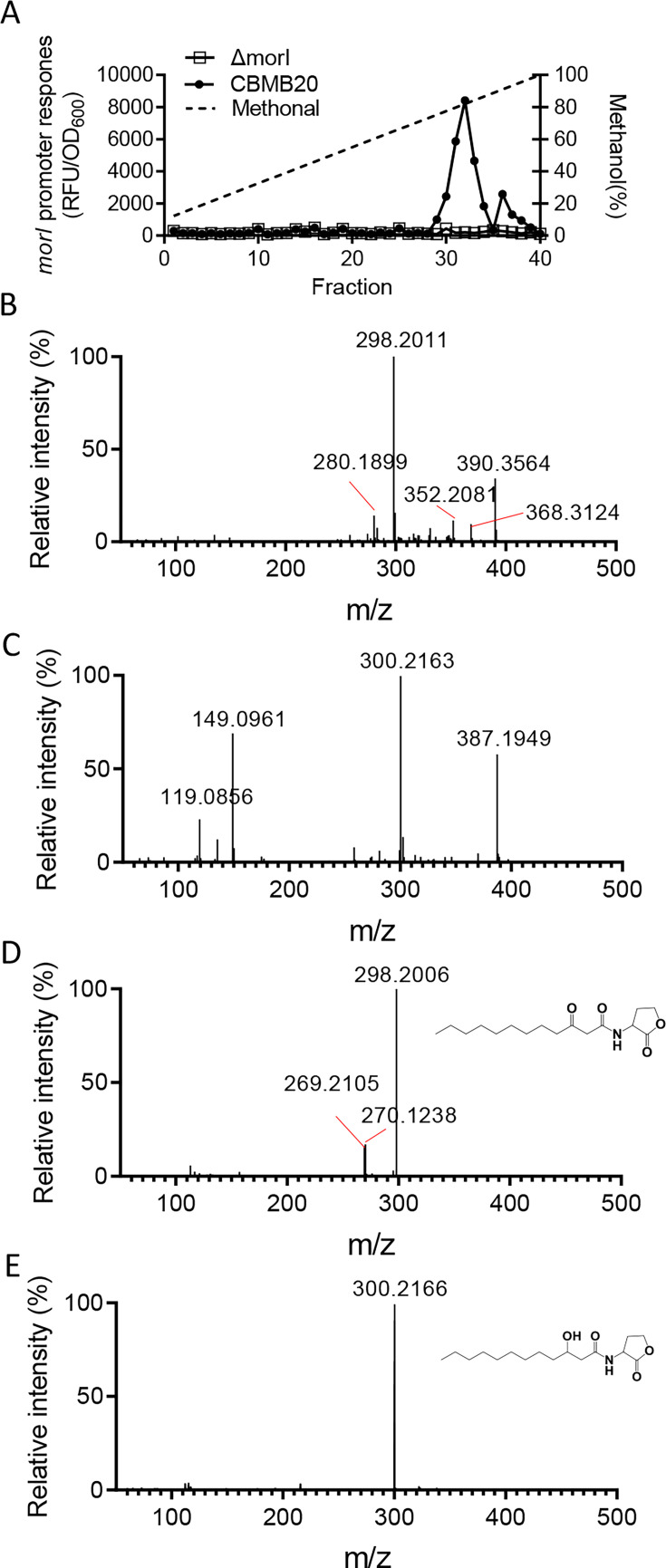
Detection and purification of *M. oryzae* CBMB20 signals. (**A**) Gradient HPLC profiles of AHLs synthesized by *M. oryzae* CBMB20 (●) and the morI mutant (□). The dashed line shows the methanol gradient. (**B and C**) Mass spectrum showing the parental ions of fractions 33 (**B**) and 36 (**C**). (**D and E**) Mass spectrum showing the parental ions of chemically synthesized 3-oxo-C12-HSL (**D**) and 3-OH-C12-HSL (**E**).

**Fig 2:**
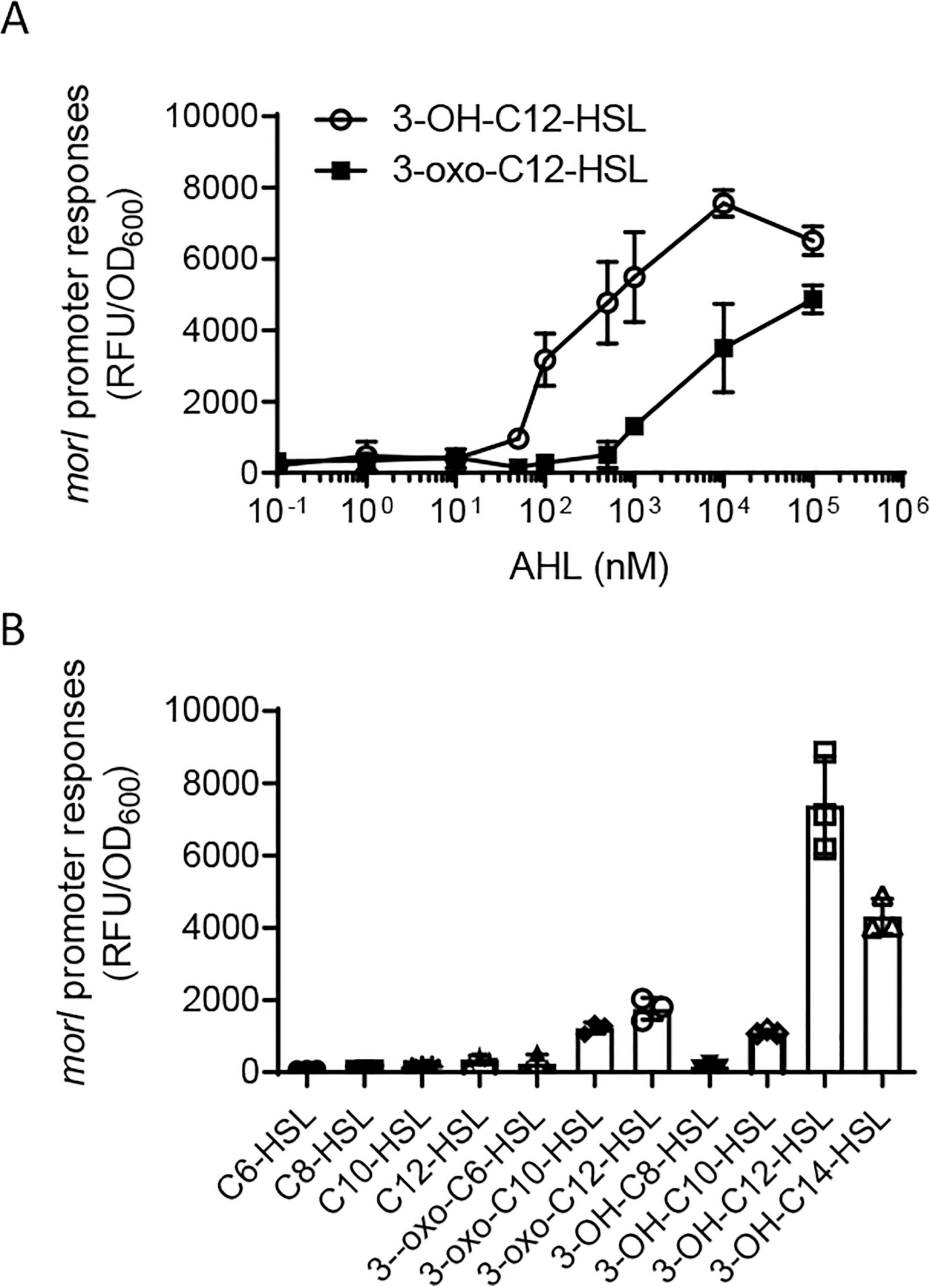
*M. oryzae* CBMB20 produces primarily 3-OH-C12-HSL and responds to the long-chain 3-hydroxyl AHLs. (**A**) Dose response of the PDQY1 reporter strain to 3-OH-C12-HSL (○) and 3-oxo-C12-HSL (■). (**B**) Relative activity of various AHLs in the induction of the reporter strain PDQY1. Each AHL signal was assayed at the final concentration of 100  nM. Error bars represent SD from three replicates.

**Fig 3:**
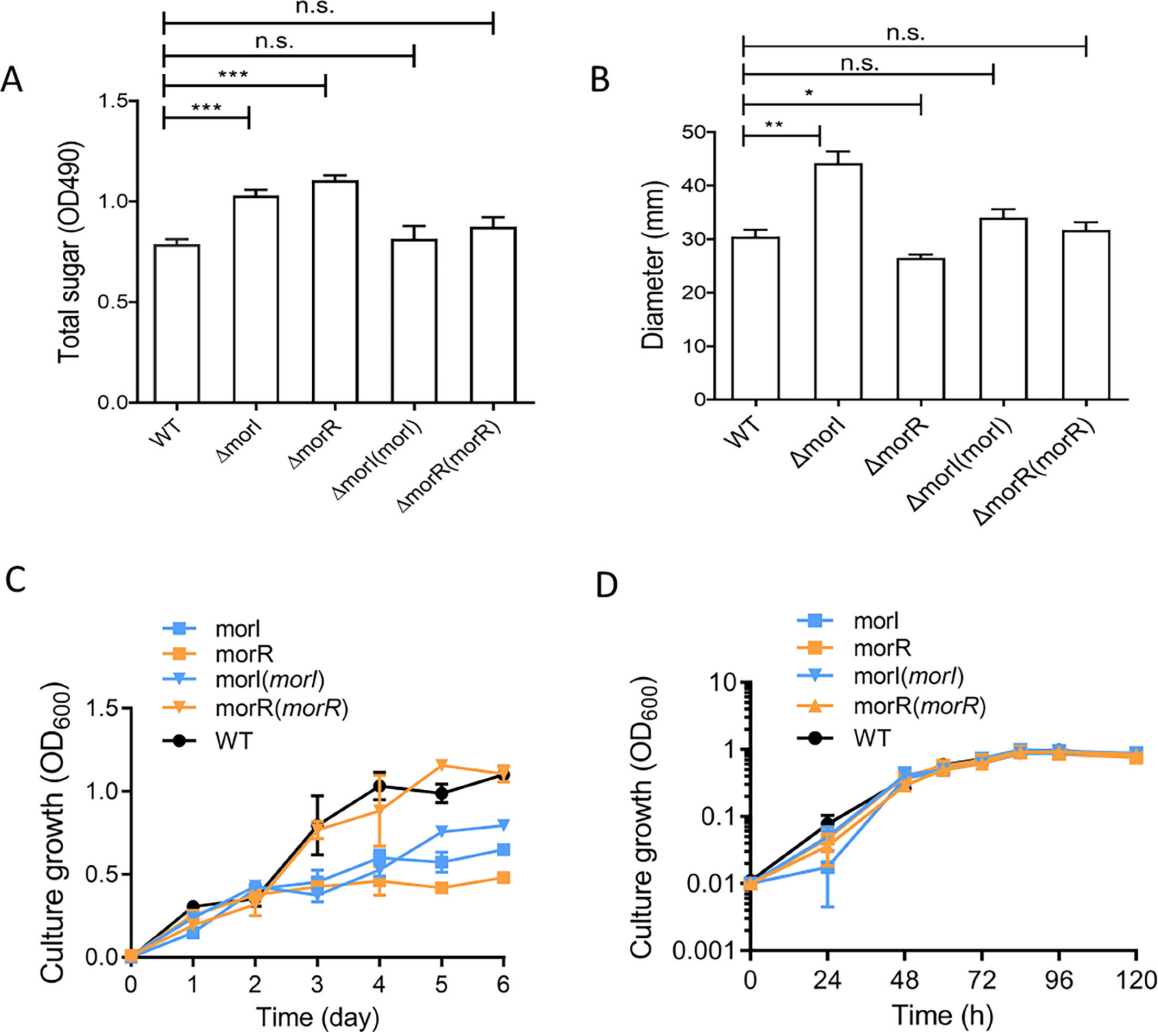
Phenotype analyses of *M. oryzae* CBMB20. (**A**) Total sugar production. (**B**) Swimming motility. (**C**) Growth curves of *M. oryzae* CBMB20 and mutants in ammonium mineral salts (AMS) minimal broth medium with 10 mM sodium succinate. (**D**) Growth curves of *M. oryzae* CBMB20 and mutants in AMS minimal broth medium supplemented with 1% methanol. The error bars indicate SDs. Statistical analysis was performed using Student’s *t* test. *, *P* < 0.05; **, *P* < 0.01; ***, *P* < 0.001; n.s., no significance compared to the indicated group.

To isolate the AHL molecules synthesized by MorI, we extracted and concentrated the 2L cell-free supernatants of strain CBMB20 using acidified ethyl acetate. The signal activity was predominantly located in the organic phase, which was then subjected to evaporation and dissolution in methanol. The extracts were tested for AHL production using the PDQY1 reporter strain, confirming the stimulation of violacein production. The methanol solution was appropriately diluted and fractionated using C18 reverse-phase high-performance liquid chromatography (RP HPLC) with a methanol gradient. The active fractions (Fig. 1A) were identified and further resolved by isocratic HPLC. High-resolution liquid chromatography tandem mass spectrometry (LC-MS/MS) analysis of these active fractions identified parent ions [M + H]^+^ of 298.2011 m/z (Fig. 1A) and 300.2163 m/z (Fig. 1C), corresponding to synthetic N-(3-oxo-dodecanoyl)-HSL (3-oxo-C12-HSL; Fig. 1D) and N-(3-hydroxydodecanoyl)-HSL (3-OH-C12-HSL; Fig. 1E) standards, respectively. Both parent ions were accompanied by a characteristic daughter ion [M + H]^+^ of 102 (Fig. S3), common to most AHLs. The HPLC retention times and mass spectra of the purified compounds were indistinguishable from commercial standards, confirming that the MorI enzyme in *M. oryzae* strain CBMB20 synthesizes two QS signals: 3-oxo-C12-HSL and 3-OH-C12-HSL.

### MorR responds to 3-OH-C12-HSL signals

In order to evaluate the biological activity of the AHL signals, we utilized the PDQY1 reporter strain to compare their signaling efficacy. The data revealed that the reporter strain was sensitive to both types of AHL signals, with 3-OH-C12-HSL demonstrating superior effectiveness, especially at lower concentrations. This implies that the MorI/R quorum-sensing system in strain CBMB20 predominantly generates and is activated by the AHL signal 3-OH-C12-HSL (Fig. 2A). To further investigate the selectivity of MorR, we examined its response to eleven distinct AHLs, each with fatty acyl chains varying in length from 6 to 14 carbons. At a final concentration of 100 nM, 3-OH-C12-HSL was the most effective activator of the PDQY1 reporter, followed by 3-OH-C14-HSL, 3-oxo-C12-HSL, 3-oxo-C10-HSL, and 3-OH-C10-HSL (Fig. 2B). The reporter strain showed minimal response to C6-HSL, C8-HSL, C10-HSL, C12-HSL, 3-oxo-C6-HSL, or 3-OH-C8-HSL (Fig. 3B). This suggests that the signal receptor MorR preferentially responds to long-chain AHLs.

### The MorI/R QS system controls a range of biological functions

In an effort to delineate the biological functions governed by the MorI/R QS system, we generated deletion mutants for both *morI* and *morR* genes, utilizing the wild-type (WT) CBMB20 strain as the control. Additionally, we created complemented strains to restore the WT phenotype. Our phenotypic analysis revealed significant changes in the mutants compared to the parental strain. The *morI* and *morR* deletion mutants exhibited a notable increase in EPS (Fig. 3A). In a semisolid motility agar assay, the ΔmorI mutant demonstrated a marked enhancement in swimming motility, whereas the ΔmorR mutant displayed a pronounced reduction in this ability (Fig. 3B). When assessing the capacity of the MorI/R QS system to utilize methanol as the sole carbon and energy source, both ΔmorI and ΔmorR mutants showed a significant decrease in growth capability (Fig. 3C). In contrast, when grown on sodium succinate without methanol, the growth of these mutants did not differ significantly from that of the WT strain (Fig. 3D). Complementation of ΔmorR and ΔmorI mutants largely restored the wild-type phenotypes (Fig. 3A, B and D), with the exception of methanol utilization, which was only partially rescued in the ΔmorI mutant (Fig. 3C). These findings underscore the pivotal roles of the MorI signal and the MorR regulatory protein in modulating these phenotypic traits.

### MorR and MorI control distinct but overlapping regulons in *M. oryzae* CBMB20

**Fig 4:**
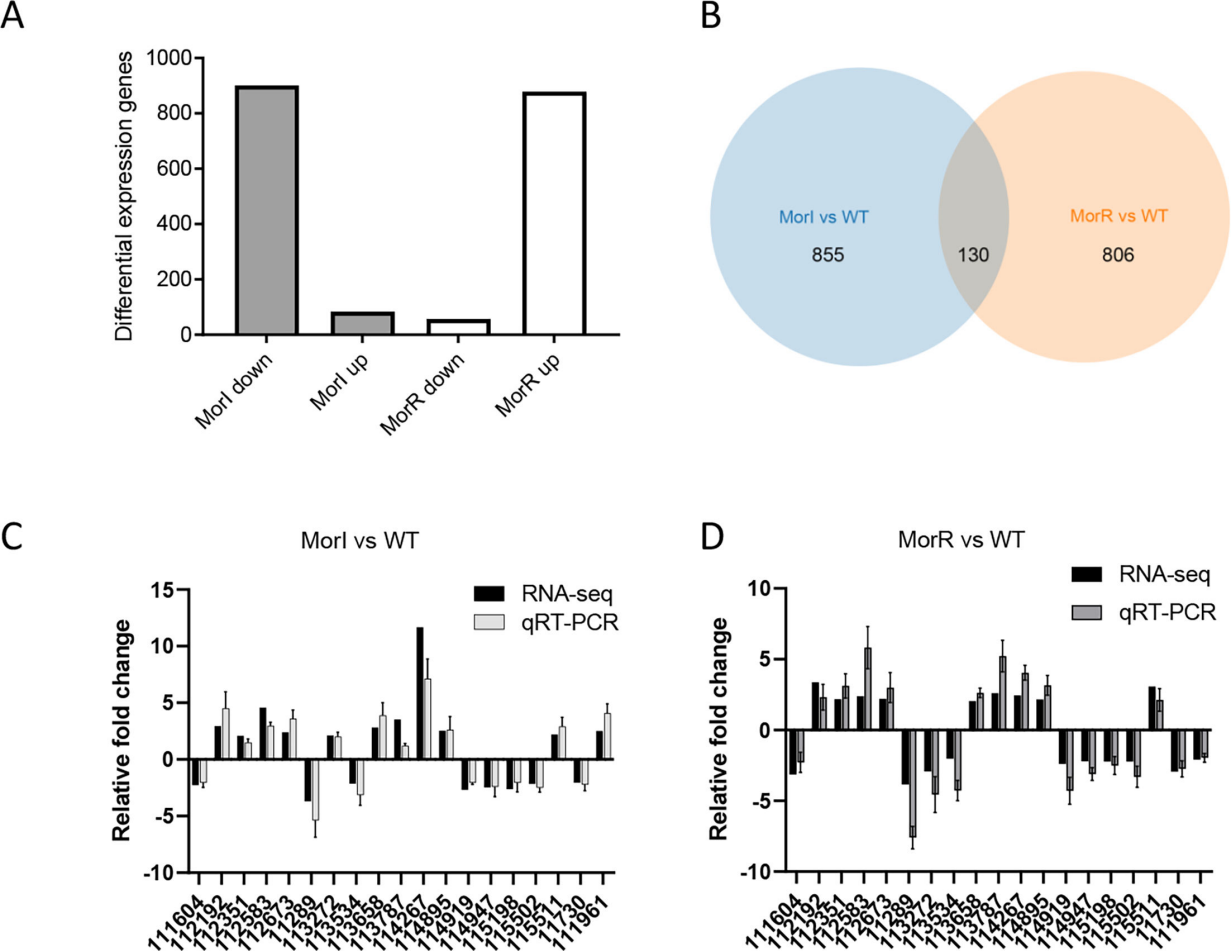
MorI and MorR regulons in *M. oryzae* CBMB20. (**A**) Transcriptome sequencing (RNA-seq) in WT, △morI, and △morR. (**B**) Venn diagram showing overlapping and specifically regulated genes by MorI and MorR, respectively, in *M. oryzae* strain CBMB20; qRT-PCR validation of MorI (**C**) and MorR (**D**) upregulated and downregulated genes unveiled by RNAseq analysis. qRT-PCR data were normalized to *recA* in WT, *morI*, and *morR*. Error bars represent SD of three replicates.

In order to delineate the regulatory scope of the MorI/R QS system, we employed transcriptome sequencing (RNA-seq) to compare the global transcriptional profiles between the WT strain CBMB20 and *morI* or *morR* deletion mutants. The genomic transcriptional profiles showed that MorR regulates the expression of 936 genes, with 878 being upregulated and 58 downregulated, as determined by a twofold change in expression levels when comparing the *ΔmorR* mutant to the WT CBMB20 with *P* value < 0.05 (Fig. 4A). Similarly, we identified 985 genes (82 upregulated and 903 downregulated) that are regulated by MorI (Fig. 4A). To substantiate the RNA-seq findings, we randomly selected 19 genes both from MorI and MorR regulon for quantitative reverse transcription-PCR (qRT-PCR) analysis (Fig. 4C and D). The qRT-PCR data from the *ΔmorI* and *ΔmorR* mutants were highly consistent with the RNA-seq results, thereby validating the transcriptome analysis (Fig. 4C and D). Inspection of RNA-seq data, we note that only 130 genes were found to be coregulated by both MorR and MorI (Table S1 and Fig. 4B). This indicates that MorR regulates the expression of numerous genes independently of its canonical AHL signal synthetase MorI.

### MorR acts as a repressor in *M. oryzae* CBMB20

**Fig 5:**
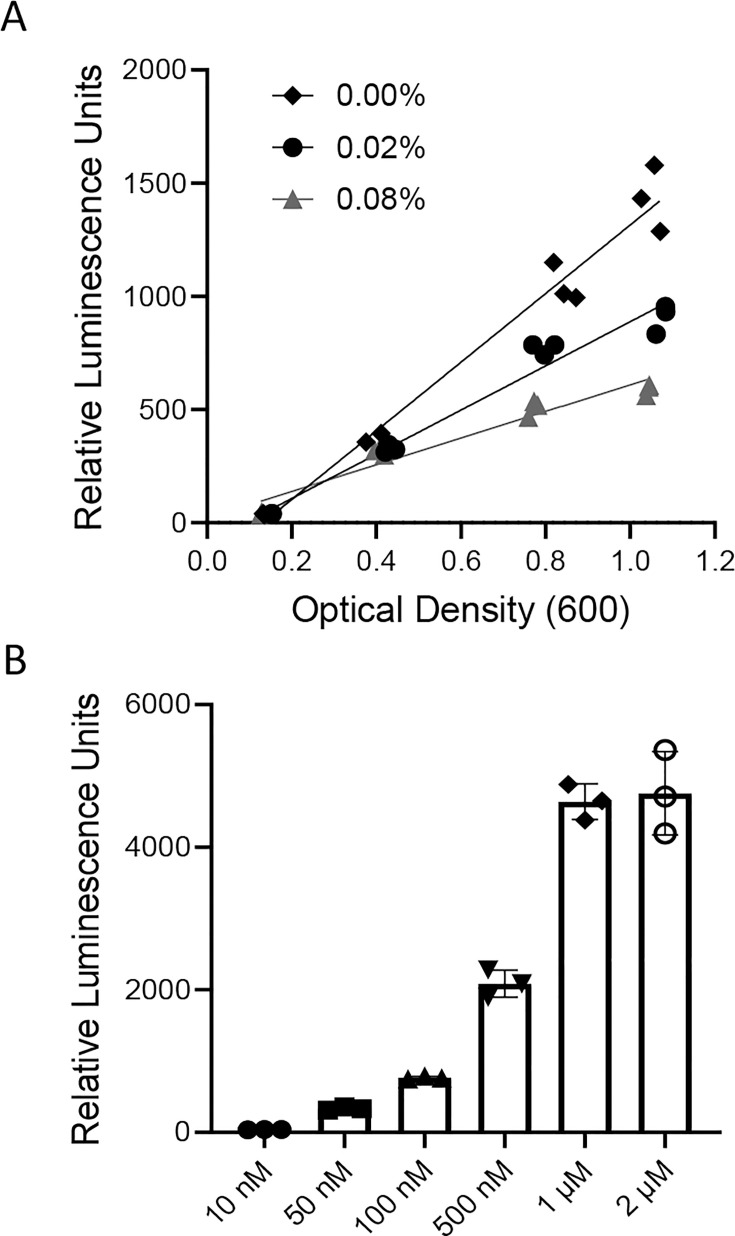
Dose-dependent repression and derepression of an *morR* translational fusion. The *E. coli* strain DH5a harboring plasmids pHFW1 and pHFW2 was grown (**A**) to different optical densities (600 nm) in the presence of 0% (◆), 0.02% (●), or 0.08% (▲) L-arabinose; or (**B**) to an optical density (600 nm) of 0.6 in the presence of 0.08% L-arabinose with 0, 50 nM, 100 nM, 500 nM, 1 µM, and 2 µM 3-OH-C12-HSL.

From the genomic transcriptional profiles above, deletion of *morR* resulted in more than 93% significant differentially expressed genes (DEGs) upregulated. Hence, we speculate that MorR acts as a repressor in *M. oryzae* CBMB20. To validate our hypothesis, we engineered an *in vivo* assay leveraging the coexpression of plasmids pHFW1 and pHFW2 within the *Escherichia coli* DH5α host strain, HF01. While pHFW1 provides the *morR* gene under control of the pBAD promoter, pHFW2 harbors a *luxABCDE* reporter gene driven by the *morI* promoter, which includes a predicted MorR-binding sequence. Cultivating strain HF01 in a glucose-rich medium devoid of L-arabinose resulted in maximum luminescence, indicating full induction (Fig. 5A). Conversely, the presence of 0.02% L-arabinose significantly reduced luminescence to approximately one-tenth of the initial level (Fig. 5B). Varied reporter activity levels were observed, inversely related to L-arabinose concentration, confirming MorR as a repressor, independent of AHL signaling.

To substantiate the role of MorR in QS via repression, we demonstrated that the biologically relevant AHL signal, 3-OH-C12-HSL, could induce derepression. Strain HF01 was cultivated under repressive conditions with 0.08% L-arabinose, and cultures were treated with synthetic 3-OH-C12-HSL at concentrations from 10 mM to 2 µM. Reporter derepression was directly proportional to the 3-OH-C12-HSL concentration (Fig. 5B). These findings suggest that AHL signaling counteracts MorR-mediated repression of *morI* gene, highlighting the dynamic interplay between QS signaling and gene regulation in *Methylobacterium* species.

## DISCUSSION

The study on *M. oryzae* CBMB20 has provided valuable insights into the role of the MorI/MorR QS system in regulating various biological functions. The identification of two QS signals, 3-oxo-C12-HSL and 3-OH-C12-HSL, synthesized by MorI, aligns with previous findings that *Methylobacterium* species utilize LuxI homologs to produce AHLs under different conditions (13). The preference of MorR for 3-OH-C12-HSL, particularly at lower concentrations, is consistent with the specificity observed in other LuxR-type receptors, which can discriminate between AHLs based on chain length and oxidation state (1–4).

QS is utilized by numerous *Methylobacterium* species (3, 21), which produce and respond to AHLs to regulate diverse cellular processes (13–17). This study identifies the MorI/MorR QS system as a key regulator of multiple functions in *M. oryzae* CBMB20, including EPS production, motility, and methanol utilization. Crucially, control of EPS production is a well-documented feature of AHL-based QS systems in many bacteria (3), including *Methylobacterium* spp. (14). However, EPS production in *Methylobacterium* is also influenced by several other factors, such as methanol availability (22), chromium (VI) concentration (23), and elevated carbon levels (24). Therefore, EPS production likely results from complex multi-factorial regulation, and the specific regulatory role of QS within this network requires further investigation. Notably, EPS from *M. extorquens* PA1 significantly alleviates plant abiotic stress and may contribute to bacterial biofilm formation. Unfortunately, under the laboratory conditions tested, we observed no discernible differences in biofilm formation between CBMB20 wild-type and *morI/morR* mutants. Interestingly, EPS yield in M. oryzae CBMB20 correlates with cellular aggregation, suggesting that QS in this strain may influence aggregation, either in laboratory settings or potentially in the plant environment.

As methylotrophs commonly associated with plants, *Methylobacterium* species utilize single-carbon compounds like methanol and methane. Intriguingly, MlaI produces the unusual unsaturated long-chain AHL molecules 7Z-C14-HSL and 2E,7Z-C14-HSL specifically when *M. extorquens* AM1 is grown on methanol (14). Furthermore, the presence of a QS system in the *M. tundripaludum* 21/22 underscores the link between QS and methylotrophy (16). This connection likely occurs through the regulation of core methylotrophic metabolism components, such as methanol dehydrogenases or downstream C1 assimilation pathways, which are crucial for niche adaptation. Characterizing additional QS systems in methylotrophs will be essential to fully elucidate their biological roles.

Transcriptomics revealed significant regulatory divergence between the synthase and receptor. While MorI and MorR co-regulate 130 genes, MorR independently governs the expression of over 800 genes. This suggests that MorR integrates signals beyond its cognate AHL. Specifically, MorR may respond to endogenous or host-derived ligands other than 3-OH-C12-HSL/3-oxo-C12-HSL, acting as a broader spectrum sensor. Notably, plants (including rice and beans) have evolved mechanisms to exploit AHL-mimic QS signals, either responding to or interfering with bacterial communication for their own benefit (25). Furthermore, MorI may influence gene expression independently of MorR. MorI-derived AHLs could potentially interact with non-canonical receptors or fulfill signaling-independent roles. Collectively, this complexity underscores that the morI/morR locus is embedded within a broader, interconnected regulatory network, where MorR serves as a key node integrating QS with other signaling inputs.

The data reveal that MorR functions as a repressor, regulating a substantial number of genes, the majority of which are upregulated upon its deletion. This indicates that MorR likely maintains basal-level gene expression under non-QS conditions. *In vivo* assays further confirmed MorR’s repressive role, which can be relieved by AHL signaling. This functionality provides a mechanism for fine-tuning gene expression in response to environmental cues. LuxR-type proteins are central components of bacterial QS, governing group behaviors and metabolism in response to signaling molecules (26). Their specific roles vary across species and ecological niches, and they often integrate into complex regulatory networks. The functional diversity within the LuxR family mirrors the wide spectrum of bacterial lifestyles and highlights the critical importance of quorum sensing in bacterial adaptation. Understanding LuxR mechanisms is therefore essential for applications in medicine, agriculture, and environmental science.

The MorI/R QS system in *M. oryzae* CBMB20 operates through a sophisticated signal-specific derepression mechanism. MorR acts as a master repressor, integrating QS (via 3-OH-C12-HSL) and potentially other signals to govern a vast regulon, including genes essential for phyllosphere colonization (motility, EPS), metabolic efficiency (methanol use), and thus, its mutualistic interaction with rice. The observed regulatory complexity and phenotypic impacts underscore the central role of this QS system in bacterial adaptation within the plant niche and highlight its potential as a focal point for enhancing the efficacy of PGPB applications. Future work should prioritize identifying key effector genes within the MorR regulon, elucidating signals beyond AHLs that influence MorR, and validating QS manipulation strategies for improved plant growth promotion.

## MATERIALS AND METHODS

### Bacterial strains and culture conditions

The plasmids and bacterial strains used in this study are listed in Table S2. *M. oryzae* CBMB20 strains were grown at 28°C in ammonium mineral salts (AMS) minimal broth medium (27) or adding 1% CH_3_OH when needed, for 5 days. *P. putida* F1 and reporter strains were cultured at 30°C in Luria-Bertani (LB) broth at a pH of 6.8 employing 50 mM 3-(N-morpholino) propane sulfonic acid (Mops) as a buffer (LB-Mops broth) (20), and *E. coli* strains were cultured at 37°C in LB broth, which consists of 10.0 g of tryptone, 5.0 g of yeast extract, and 10.0 g of NaCl per liter. The swimming motility soft agar contains 10.0 g Bacto-Tryptone, 5.0 g bacteriological yeast extract, and 2.0 g bacteriological agar per liter. Antibiotics were used as needed at the following concentrations: 100 µg/mL ampicillin, 100 µg/mL kanamycin, and 50 µg/mL gentamicin. AHL derivatives were used as necessary at a final concentration of 100 nM, unless otherwise indicated.

### Mutant and plasmid construction

Mutants with deletion of MorI and MorR were derived from *M. oryzae* CBMB20 by DNA fusion fragments as previously described (28). Briefly, an upstream DNA fragment and a downstream DNA fragment of the MorI/R QS system genes of about 500–800 bp flanking the gene of interest with *M. oryzae* CBMB20 genomic DNA as the template were PCR amplified and fused with gentamicin fragments by standard enzyme-based techniques. PCR fragments transformation of *M. oryzae* CBMB20 was performed similarly to that described previously. The complemented strains were derived by in *trans* expression of corresponding WT genes in the MorI/R QS system deletion mutants, respectively. The coding sequences of MorI/R genes were amplified by PCR using specific primer pairs (Table S2) that were cloned into the pBBR1-MCS-2 by standard techniques. The resultant constructs were transformed into *E. coli* DH5a and the MorI/R QS system deletion mutants. All deletion mutations and complemented mutants were confirmed by PCR and DNA sequencing.

The MorR-PmorI-mCherry reporter plasmid (pDQY1) was constructed by fusing the *morI* promoter with the ORF of the *mCherry* gene, which, together with the MorR coding sequence, was cloned into the expression vector as previously described (29). Briefly, the DNA fragments containing the predicted MorI promoter and the MorR ORF were amplified by the corresponding primers listed in Table S2 using *M. oryzae* CBMB20 genomic DNA as the template. The linearized pBBR-mCherry fragment was generated by PCR amplification using the plasmid pLL1 as the template (20). Two DNA fragments contain at least 25 bp of homologous overlap, which is required for *E. coli* DH5a-mediated assembly. Transformants were selected on LB plates containing 50 mg/mL gentamicin and confirmed by DNA sequencing with the MorR-Check-F and MorR-Check-R primers (Table S2). The confirmed construct was electroporated into *P. putida* F1 to generate the reporter strain PDQY1. The primers used for the construction of the reporter strains are listed in Table S2. A similarity-based method was used for the construction of plasmids pHFW1 and pHFW2. We amplified the *morR* gene using PCR primers; the *morR* fragment was inserted into the similarly digested expression vector, pJN105 (30) resulting in plasmid pHFW1. The *morI* promoter was inserted into plasmid pMS402 (31) to create pHFW1 (Table S1).

### Bioassay of AHL signals

The AHL bioassay was performed with the reporter strain DQY1 using the following protocol. The bacterial cell-free culture supernatants of OD_600_ = 1.5 were prepared by centrifugation to remove cells, which were then extracted twice with an equal volume of acidified ethyl acetate (0.1 mL of glacial acetic acid/L). The dried samples were reconstituted in 200 µL of methanol. Five microliter ethyl acetate extracts were added to 16 mL glass tubes, and ethyl acetate was removed by evaporation under a gentle stream of N_2_ gas. Overnight culture of the reporter strain DQY1 was diluted to an OD_600_ of 0.05 and was added to the tubes containing the signal solution in a final volume of 1.0 mL LB-Mops broth. The tubes were then incubated for 24 hours at 28°C with shaking, and then fluorescence was measured as described previously (20).

### Purification of AHLs from *M. oryzae* CBMB20

To purify the AHL signals synthesized by MorI, *M. oryzae* CBMB20 was inoculated in 1 L of AMS minimal broth medium adding 1% CH_3_OH and cultured until it reached an OD_600_ of about 1.5. The bacterial cells were removed by centrifugation, and supernatants were extracted with two equal volumes of 0.1% acidified ethyl acetate. The concentrated extracts were fractionated by the C18 RP HPLC column, as described previously (20). Each 2 mL fraction was collected and assayed by using the reporter strain DQY1, as described in the previous section. The bulk of the active fractions was collected, dried, and separated by isocratic HPLC with 80% methanol. Fractions were collected, dried, and tested as described in the text. Two purified active fractions and synthetic N-(3-hydroxydodecanoyl)-HSL and N-(3-hydroxytetradecanoyl)-HSL were analyzed by LC-MS/MS in a Waters Acquity ultraperformance liquid chromatography C18 RP column (1.7 mm, 2.1 mm by 30 mm) with a Thermo Linear Trap Quadrupole Orbitrap MS System (collision energy = 40 eV and cone = 35 V) using the same approach.

### RNA preparation, RNA-seq, data analysis, and qRT-PCR

Total RNA was extracted with Eastep Super Total RNA extraction kit (Promega, Madison, WI, USA) from 1 mL cultures of *M. oryzae* CBMB20 strains in NB with 1% CH_3_OH broth when the OD600 reached about 1.5. Ribosomal RNAs were removed from the prepared total RNA samples with the RiboZero rRNA removal kit (for Gram-negative bacteria; Illumina, Madison, WI, USA). cDNA library preparation and RNA sequencing were performed by Novogene (Beijing, China) using Illumina HiSeq 2500 single-end reads. Clean reads were obtained by removing adaptors, unknown nucleotides, and low-quality reads. In addition, clean reads were aligned to the *M. oryzae* CBMB20 genome (NCBI Taxonomy ID 693986, GenBank accession no. GCA_000757795.1 and Refseq GCF_000757795.1), using Bowtie2 v.2.2.3 (32). For expression quantification, the number of read pairs aligned to each gene was counted using the featureCounts tool from the subread package v.0.30 (33). Additionally, the fragments per kilobase of transcript per million mapped reads method was used to normalize the level of gene expression (34). Moreover, DESeq2 was used for differential expression analysis (35), using the Benjamini-Hochberg adjustment for multiple comparisons and a false-discovery rate of 0.05. The DEGs were then subjected to enrichment analysis through Gene Ontology and Kyoto Encyclopedia of Genes and Genomes pathway analyses. RNA samples were reverse transcribed into cDNA using HiScript III RT SuperMix for quantitative PCR (qPCR; Vazyme). qRT-PCR analyses were performed with ChamQ Universal SYBR qPCR master mix (Vazyme). The gene expression level of *recA* was used as a control for qRT-PCR analysis. The primers used in qRT-PCR analysis are listed in Table S2. The absolute values of relative expression of target genes were calculated using the threshold cycle (2^–△△CT^) method, as described previously (36). The RNA samples were extracted twice, and each time, qRT-PCR was performed in triplicate.

### Exopolysaccharide assays

Exopolysaccharide (EPS) was isolated from *M. oryzae* CBMB20 culture supernatants following an established ethanol precipitation protocol with modifications (20). The bacterial culture was harvested during mid-logarithmic phase growth in AMS minimal medium. For EPS extraction, 2 mL of cell-free supernatant was combined with three volumes of pre-chilled absolute ethanol (6 mL) and subjected to overnight precipitation at −20°C. The precipitated polysaccharides were pelleted by centrifugation at 16,800 × *g* for 30 min (4°C), followed by resuspension in 100 µL ultrapure water (Milli-Q system). Total sugar was quantified using the phenol-sulfuric acid microassay. Specifically, 50 µL aliquots of the EPS solution were mixed with 30 µL of 5% (wt/vol) phenol solution and 150 µL concentrated sulfuric acid in 96-well microplate format. After thorough vortex mixing, absorbance at 490 nm was recorded using a Biotek H1 microplate reader, with glucose serving as the calibration standard. All experimental measurements were performed in triplicate.

### Swimming assay

To assess bacterial swimming motility, soft agar plates were prepared by mixing 0.5% agar with nutrient broth, and an overnight bacterial culture was spotted onto the surface. The plates were incubated at 37°C for 6–24 hours, after which the diameter of halos formed around the inoculation point, indicating the extent of bacterial swimming, was observed and measured. Larger halos suggest greater motility. The culture was confirmed to be viable, and conditions were suitable for motility. The experiments were repeated at least twice in triplicate.

### Statistical analysis

All of the experiments were repeated at least twice in triplicate or as otherwise indicated. Statistical significance was determined with a two-tailed *t* test for comparison between two treatments and one-way analysis of variance for comparison between multiple treatments, where a *P* value of 0.05 was considered statistically significant. *P* values for all respective analyses are indicated in the figure legends.

## Data Availability

The raw data from transcriptome analyses were deposited in the NCBI Sequence Read Archive (SRA) under accession no. PRJNA1156340.

## SUPPLEMENTAL MATERIAL

Supplemental figures spectrum.02117-25-s0001.docx Fig. S1 to S3.

Table S1 spectrum.02117-25-s0002.xlsx Genes co-regulated by MorR and MorI.

Table S2 spectrum.02117-25-s0003.docx Strains, plasmids, and primers used in this study.

ASM does not own the copyrights to Supplemental Material that may be linked to, or accessed through, an article. The authors have granted ASM a non-exclusive, world-wide license to publish the Supplemental Material files. Please contact the corresponding author directly for reuse.
