# Supplementary material for: The role of MorI/MorR quorum sensing in Methylobacterium oryzae CBMB20: modulating bacterial functions for enhanced adaptability
Source: Microbiol Spectr. 2025 Sep 12;13(10):e02117-25. doi: 10.1128/spectrum.02117-25 (PMC12502750; doi:10.1128/spectrum.02117-25)
Supplement: Supplemental figures — Fig. S1 to S3. [file spectrum.02117-25-s0001.docx]

**Supplementary Materials**

**Title：**The Role of MorI/MorR Quorum Sensing in Methylobacterium oryzae CBMB20: Modulating Bacterial Functions for Enhanced Adaptability

**Authors:** Qiying Deng1*, Yue Zheng2, 3*, Huagui Gao1, Haofang Wu1, Enyu Shi1, Mengmeng Cheng1, Lisheng Liao1^#^

**Content:** Supplementary Figure S1 – S3

**
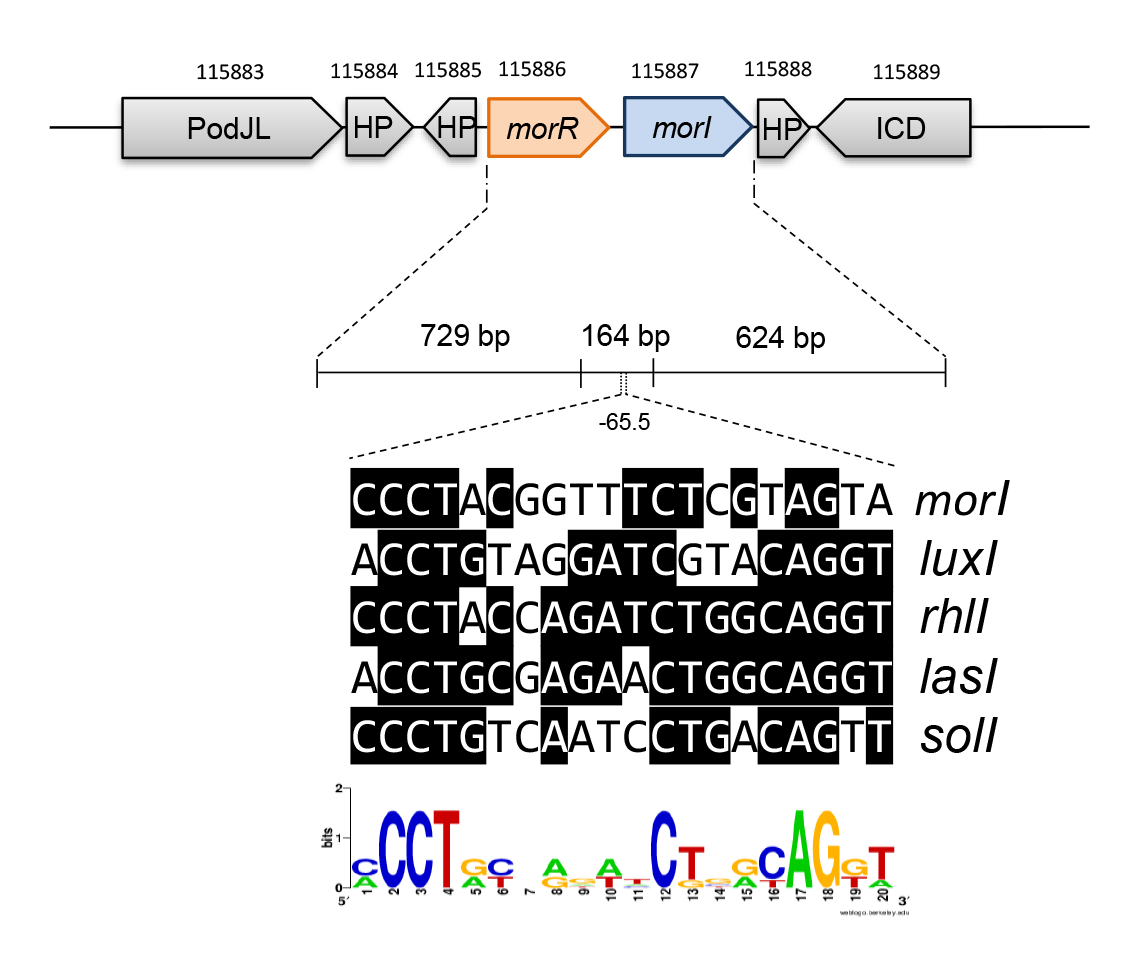
**

**Fig. S1** MorI promoter analysis. Sequence alignment of the putative MorR-binding site in *morI* promoter with inverted repeats found in promoter regions of *luxI*-type gene family members from *V. fischeri* ES114 (*luxI*), *P. aeruginosa PAO1* (*rhlI* and *lasI*), *R. solanacearum* (*solI*). The sequence logo was constructed using WebLogo (<http://weblogo.berkeley.edu/>).


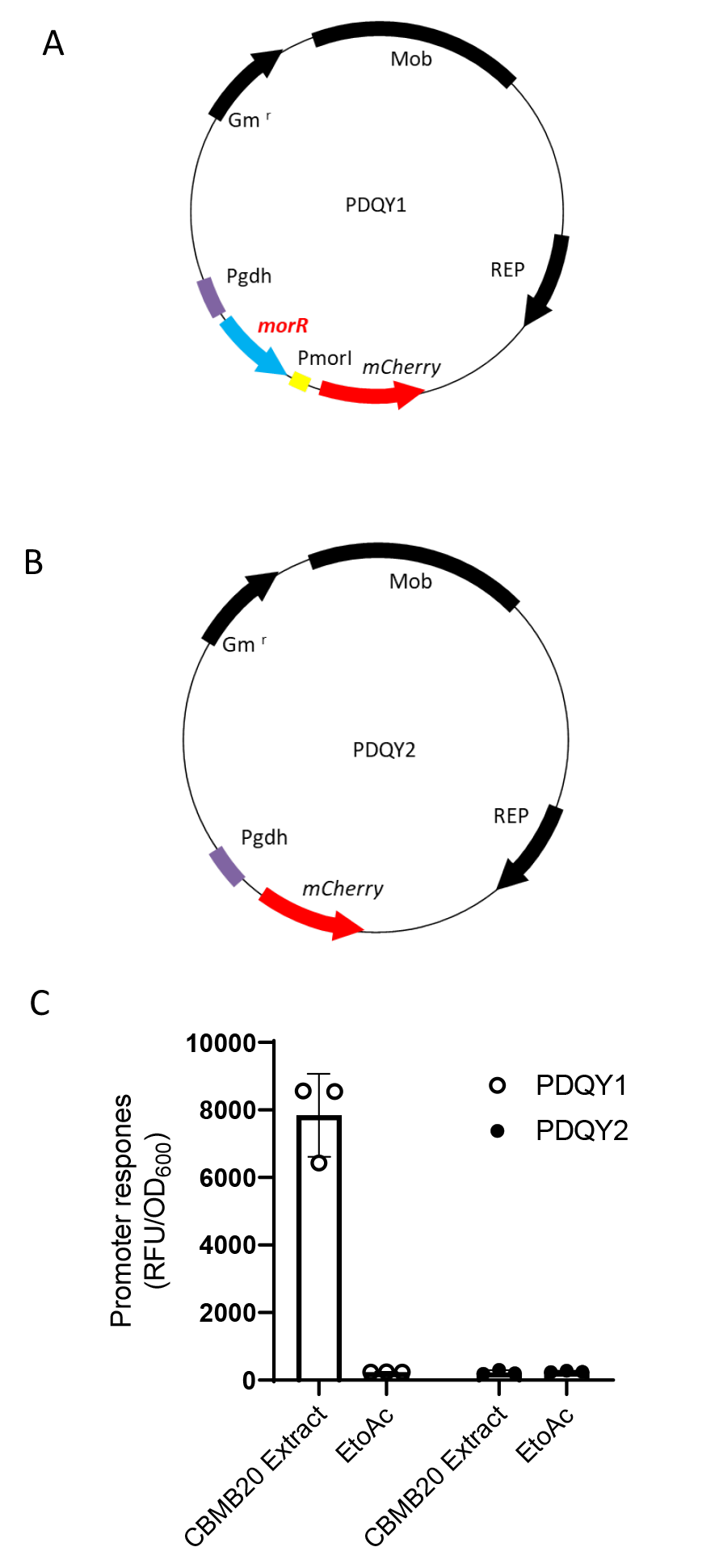


**Fig. S2** Construction of reporter strains for AHL QS signals in *M. oryzae* CBMB20. Genetic map of the reporter construct pDQY1 (A) and pDQY2 (B). Pgdh is a strong promoter for driving *morR* gene expression. Bioassay testing for *M. oryzae* CBMB20 culuture extract and EtoAc as control (C).


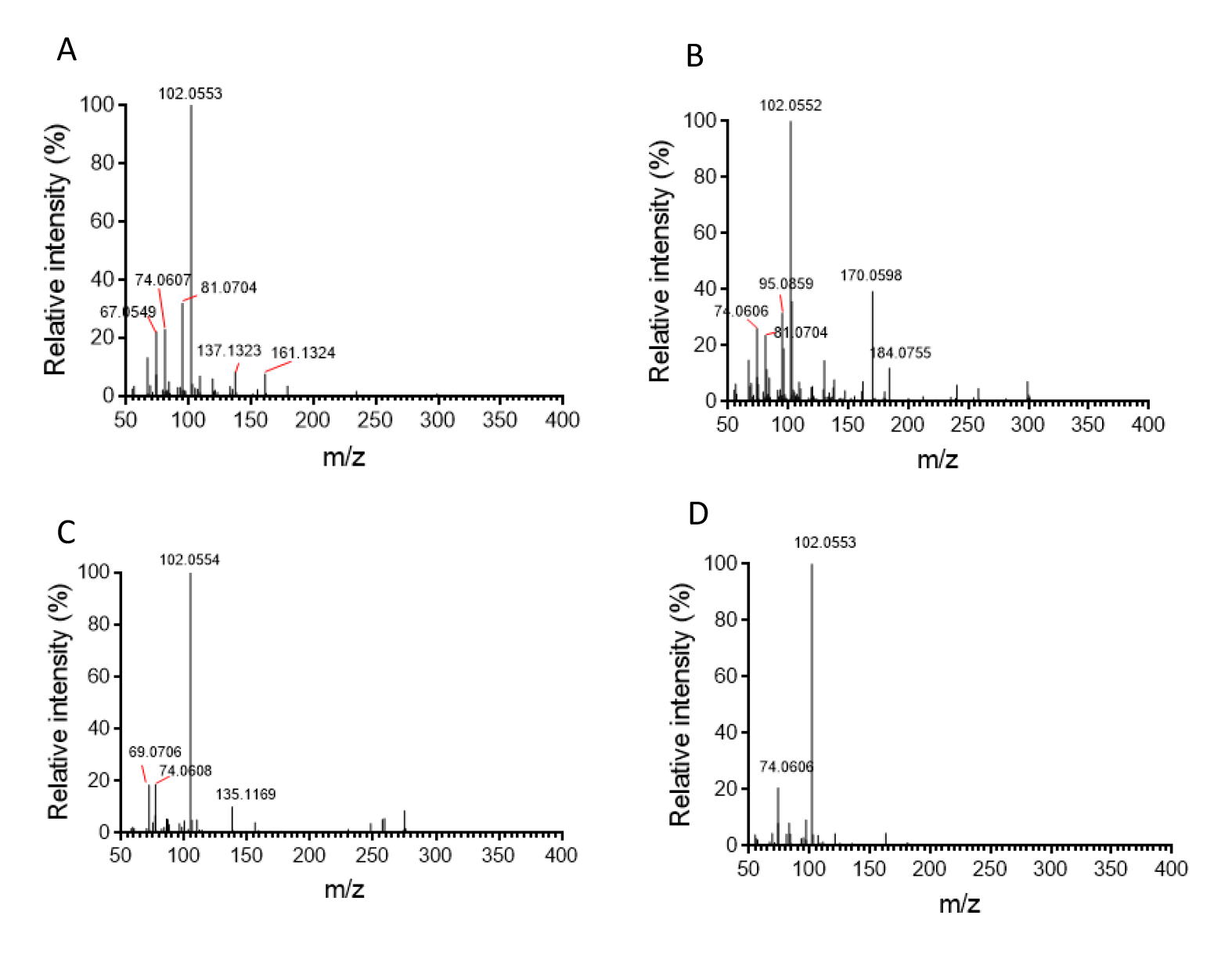


**Fig. S3** Purification and characterization of the AHL signals produced by MorI. MS/MS daughter scan of the m/z 298 (M+H) and m/z 300 (M+H) ion from natural product fractions. (A) and (B), MS/MS daughter ion analysis of the parent ions m/z 298 (M+H) and m/z 300 (M+H) shown in Figure 2, respectively. (C) and (D), MS/MS daughter ion analysis of chemically synthesized 3-oxo-C12-HSL and 3-OH-C12-HSL, respectively.
