# Supplementary material for: The role of MorI/MorR quorum sensing in Methylobacterium oryzae CBMB20: modulating bacterial functions for enhanced adaptability
Source: Microbiol Spectr. 2025 Sep 12;13(10):e02117-25. doi: 10.1128/spectrum.02117-25 (PMC12502750; doi:10.1128/spectrum.02117-25)
Supplement: Table S2 — Strains, plasmids, and primers used in this study. [file spectrum.02117-25-s0003.docx]

Table S2. Strains, plasmids and primers used in this study

| **Strain and plasmid** | | **Relevant genotype/phenotype** | | **Source** |
| --- | --- | --- | --- | --- |
| **Strains** |  | |  | |
| *M. oryzae* |  | |  | |
| CBMB20 | Wild-type isolated from freshwater，Pb^R^ | | (1)(2) | |
| Δ*morI* | The *morI* gene delection mutant, Gen^R^ | | This work | |
| Δ*morR* | The *morR* gene delection mutant, Gen^R^ | | This work | |
| Δ*morI*(*morI*) | Complemented mutant, Gen^r^, Kan^r^, | | This work | |
| Δ*morR*(*morR*) | Complemented mutant, Gen^r^, Kan^r^, | | This work | |
| *P. putida* |  | |  | |
| F1 | Wild-type isolated from a polluted water | | (3)(4) | |
| PDQY1 | Carried pDQY1 plasmid，Gm^R^ | | This work | |
| PDQY2 | Carried pDQY2 plasmid，Gm^R^ | | This work | |
| *E. coli* |  | |  | |
| DH5α | F^-^φ80d *lac*ZΔM15 Δ (*lac*ZYA-*arg*F) U169 *end A1 rec*A1 *hsd*R17 (r_k_^-^ , m_k_^+^) *sup*E44λ-*thi-*1-*gyr*A96 *rel*A1 *pho*A | | TRANS | |
| **Plasmids** |  | |  | |
| pBBP_gdh_ | pBBR1MCS-5 with RPA0944 promoter between KpnI and XhoI sites, Gm^R^ | | (5) | |
| pLL1 | pBBRPgdh with *hirR* gene, *hirR* downstream region and *mCherry*, Gm^R^ | | (6) | |
| pDQY1 | pBBRPgdh with *morR* gene, *morR* downstream region and *mCherry*, Gm^R^ | | This work | |
| pDQY2 | pBBRPgdh with *morR* downstream region and *mCherry,* Gm^R^ | | This work | |
| PBBR1-MCS2/5 | Broad-host-range cloning vector, Km^R^/Gm^R^ | | (7) | |
| pJN105 | araC-P_BAD_ cassette cloned in pBBR1MCS-5, gentamicin resistance, Gm^R^ | | (8) | |
| pMS402 | Expression reporter plasmid with the promoterless *luxCDABE* gene; Kn^R^, Tmp^R^ | | (9) | |
| pHFW1 | pJN105 with *morR* gene, coding sequence cloned into pJN105 | | This study | |
| pHFW2 | *morR::luxABCDE* gene fusion in pMS402 | | This study | |
|  |  | |  | |
| **Primers** |  | |  | |
| PBBR-MorI F | CATATTGTTCCGCATTCATCTGCACCCTCCTCCTCGCTGCAGATGATTTTCAGTTC | | For linear pBBPgdh forward primer | |
| PBBR-MorI R | ACGATATGGATCACCGCTCCCTCCTCCTCGCTGCAGATGATTTTCAGTTC | | For linear pBBPgdh reverse primer | |
| MorI F | ACTGAAAATCATCTGCAGCGAGGAGGAGGGTGCAGATGAATGCGGAACAATATGCG | | *morI* gene forward primer | |
| MorI R | AGCGAGGAGGAGGGAGCGGTGATCCATATCGTCAC | | *morI* gene reverse primer | |
| MorI Check F | CCGTAAGTGCGCTGTTCCAG | | pDQY2 detection forward primer | |
| MorI Check R | TGTCCGATGTCCTGCCTCAG | | pDQY2 detection downstream primer | |
| PBBR-MorR F | TCGAATGCGGCATCTGCACCCTCCTCCTCGCTGCAGATGATTTTCAGTTC | | For linear pBBPgdh-*mCherry* forward primer | |
| PBBR-MorR R | GATCCATATCGTCACCCCCAGGAGGAAAAACATATGGTGAGCAAGGGCGA | | For linear pBBPgdh-*mCherry* reverse primer | |
| MorR F | CCCTTGCTCACCATATGTTTTTCCTCCTGGGGTGTGACGATATGGATCACG | | *morR* gene forward primer | |
| MorR R | ATCATCTGCAGCGAGGAGGAGGGTGCAGATGCCGCATTCGAAGCACCTGG | | *morR* gene reverse primer | |
| MorR Check F | ATACCGAAATCGCCGGCCTC | | pDQY1 detection forward primer | |
| MorR Check R | CCAAGCGCGCAATTAACCCTC | | pDQY1 detection reverse primer | |
| MorI F1 | TCAATCTGGTGGCGACCTA | | *morI* gene upstream primer | |
| MorI R1 | TTTCCACGGTGTGCGTCCTTCGGTCCAGCCTTTCTCT | | *morI* gene upstream primer | |
| MorI F2 | AAATTGTCACAACGCCGCCTGGTGGCTTGAGGATGATG | | *morI* gene downstream primer | |
| MorI R2 | CAGAAGGTCTACGAGGAGGA | | *morI* gene downstream primer | |
| MorI Gen F | AGAGAAAGGCTGGACCGAAGGACGCACACCGTGGAAA | | gentamicin gene forward primer | |
| MorI Gen R | CATCATCCTCAAGCCACCAGGCGGCGTTGTGACAATTT | | gentamicin gene reverse primer | |
| MorR F1 | ACTATCTCGTGGCCATCGTG | | *morR* gene upstream primer | |
| MorR F2 | TTTCCACGGTGTGCGTCCAACACGCTGAAGGTCCTGT | | *morR* gene upstream primer | |
| MorR R1 | AAATTGTCACAACGCCGCCAGCTCGATACCGTCAACC | | *morR* gene downstream primer | |
| MorR R2 | ACATCATCCTCAAGCCACC | | *morR* gene downstream primer | |
| MorR Gen F | ACAGGACCTTCAGCGTGTTGGACGCACACCGTGGAAA | | gentamicin gene forward primer | |
| MorR Gen R | GGTTGACGGTATCGAGCTGGCGGCGTTGTGACAATTT | | gentamicin gene reverse prime | |
| MorI Check 1 | TATGGTCATGGGCATCACGG | | *ΔmorI mutant* detection forward primer | |
| MorI Check 2 | CTAAGTTCTCCGCGACCCTG | | *ΔmorI mutant* detection reverse primer | |
| MorR Check 1 | CGTCTGTCATCCCGTTGAA | | *ΔmorR mutant* detection forward primer | |
| MorR Check 2 | TTCGGTCCAGCCTTTCTCT | | *ΔmorR mutant* detection reverse primer | |
| 16s F | CATCATTCAGTTGGGCACTCTAGGG | | 16srDNA gene forward primer | |
| 16s R | CCACTGTCACCGCCATTGTAGC | | 16srDNA gene reverse primer | |
| 11730 F | GTGCTGAAACCCTACCTGGATTCG | | Ga0069285_11730 gene forward primer | |
| 11730 R | GAGATGCGCGGTGCCGTAATAG | | Ga0069285_11730 gene reverse primer | |
| 115511 F | GCTGACCCGCTTCACCGATTAC | | Ga0069285_115511 gene forward primer | |
| 115511 R | GCTGATGCACCACCTTCACCAAG | | Ga0069285_115511 gene reverse primer | |
| 115502 F | GCAACCAGACGCAGTTCATCCTC | | Ga0069285_115502 gene forward primer | |
| 115502 R | GCTTGAGCTTGCCGTAGAGATCC | | Ga0069285_115502 gene reverse primer | |
| 115198 F | GCAGCGCCTTCACGATCAAGAG | | Ga0069285_115198 gene forward primer | |
| 115198 R | GCAGCGCCTTCACGATCAAGAG | | Ga0069285_115198 gene reverse primer | |
| 114947 F | GCAGGAGATCGCCGACAAGTTC | | Ga0069285_114947 gene forward primer | |
| 114947 R | GAAGGCCAGCCGGACATACATG | | Ga0069285_114947 gene reverse primer | |
| 114919 F | CCTTCGAGGAGCTGATCCAGGAG | | Ga0069285_114919 gene forward primer | |
| 114919 R | AGATTTGTGCGGCGGGAACTTG | | Ga0069285_114919 gene reverse primer | |
| 114895 F | AGGTCGATCACGAGGTCTTCTGG | | Ga0069285_114895 gene forward primer | |
| 114895 R | TGATGTCGCGGAACACGATGAC | | Ga0069285_114895 gene reverse primer | |
| 114267 F | TCATGGACCTGTCGGTGCAG | | Ga0069285_114267 gene forward primer | |
| 114267 R | TTGTTCATCTCGGCGACCCG | | Ga0069285_114267 gene reverse primer | |
| 113787 F | GACCTGGTGCTCGACAACAAGAC | | Ga0069285_113787 gene forward primer | |
| 113787 R | CTGGCTCTCGCGCTTGATGAAC | | Ga0069285_113787 gene reverse primer | |
| 113658 F | CCTCGTGCAGGGTCATTACG | | Ga0069285_113658 gene forward primer | |
| 113658 R | TCACGCACCTCCGAATCCAT | | Ga0069285_113658 gene reverse primer | |
| 113534 F | GCTGCCATCCTCCTTCTTATGCC | | Ga0069285_113534 gene forward primer | |
| 113534 R | AATTTGCGACCCTTCAGTCCAGTAC | | Ga0069285_113534 gene reverse primer | |
| 113272 F | GTGATGGTGGTGCTGTCGTTCC | | Ga0069285_113272 gene forward primer | |
| 113272 R | TCGGTGAAATCGGGGATCTCCTC | | Ga0069285_113272 gene reverse primer | |
| 11289 F | GGGACACCAACCAGGAAATCGG | | Ga0069285_113272 gene forward primer | |
| 11289 R | CATCCACCAACGCACCAGCTTC | | Ga0069285_11289 gene reverse primer | |
| 112673 F | GACATCGACGATCCCAGGAC | | Ga0069285_113272 gene forward primer | |
| 112673 R | ATGATGATCAGCGGGTGCG | | Ga0069285_112673 gene reverse primer | |
| 112583 F | TCGGACACGGCATCATCATC | | Ga0069285_112583 gene forward primer | |
| 112583 R | TGAAGCTCGGTATCCGCCA | | Ga0069285_112583 gene reverse primer | |
| 112351 F | CGCTGTTCCACCACTTCCCAAG | | Ga0069285_112351 gene forward primer | |
| 112351 R | CGGCGTCGATATCTGCGTCAAG | | Ga0069285_112531 gene reverse primer | |
| 112192 F | ATGGATGCCGAGGGTGTCTGTC | | Ga0069285_112192 gene forward primer | |
| 112192 R | GCAGGGTCGGCTGGAGATAGAC | | Ga0069285_112192 gene reverse primer | |
| 111604 F | ACGATAGCCATAGCAATGAGCAGAC | | Ga0069285_111604 gene forward primer | |
| 111604 R | CGACGAAATTTACGCCGATGGTTG | | Ga0069285_11604 gene reverse primer | |

SUPPLEMENTAL REFERENCES

1. Madhaiyan M, Poonguzhali S, Ryu J, et al. (2006). Regulation of ethylene levels in canola (Brassica campestris) by 1-aminocyclopropane-1-carboxylate deaminase-containing Methylobacterium fujisawaense. Planta 224:268-78.

2. Lee MK, Chauhan PS, Yim WJ. (2011). Foliar colonization and growthpromotion of red pepper (Capsicum annuumL.) byMethylobacterium oryzaeCBMB20. Journal of Applied Biological Chemistry 2:120-125.

3. Luu RA, Schneider BJ, Ho CC, et al. (2013). Taxis of Pseudomonas putida F1 toward Phenylacetic Acid Is Mediated by the Energy Taxis Receptor Aer2. Applied and Environmental Microbiology 79:2416-2423.

4. Gibson DT, Hensley M, Yoshioka H, et al. (1970). Formation of (+)-cis-2,3-dihydroxy-1-methylcyclohexa-4,6-diene from toluene by Pseudomonas putida. Biochemistry 9:1626-30.

5. McKinlay JB, Harwood CS. (2010). Carbon dioxide fixation as a central redox cofactor recycling mechanism in bacteria. Proc Natl Acad Sci U S A 107:11669-75.

6. Liao L, Schaefer AL, Coutinho BG, et al. (2018). An aryl-homoserine lactone quorum-sensing signal produced by a dimorphic prosthecate bacterium. Proceedings of the National Academy of Sciences 115:7587-7592.

7. Kovach ME, Elzer PH, Hill DS, et al. (1995). Four new derivatives of the broad-host-range cloning vector pBBR1MCS, carrying different antibiotic-resistance cassettes. Gene 166:175-6.

8. Newman JR, Fuqua C. 1999. Broad-host-range expression vectors that carry the L-arabinose-inducible *Escherichia coli* araBAD promoter and the araC regulator. Gene 227:197-203.

9. Duan K, Dammel C, Stein J, Rabin H, Surette MG. 2003. Modulation of Pseudomonas aeruginosa gene expression by host microflora through interspecies communication. Mol Microbiol. 50(5):1477-91.
